# Tumor immunology meets oncology (TIMO) XVI, July 07–09 2022 in Halle/Saale, Germany

**DOI:** 10.1007/s00262-023-03468-6

**Published:** 2023-06-22

**Authors:** Barbara Seliger

**Affiliations:** 1 Institute for Translational Immunology, Brandenburg Medical School “Theodor Fontane”, Hochstraße 29, 14770 Brandenburg, Germany; 2 Medical Faculty, Martin-Luther-University Halle-Wittenberg, Magdeburger Straße 02, 06112 Halle (Saale), Germany; 3 Fraunhofer Institute for Cell Therapy and Immunology, 04103 Leipzig, Germany

**Keywords:** Immune escape, Immunotherapy, Immune resistance, Tumor microenvironment, Targeted therapy, TIMO 2022

## Abstract

During the TIMO meeting 2022, national and international scientists as well as clinicians gave novel insights as well as perspectives into basic and translational tumor immunology.

## Introduction

The yearly “Tumor immunology meets oncology (TIMO)” meeting did bring together at the Steintor Variety in Halle, Germany, experts in basic tumor immunology as well as in immune oncology to give insights into immune escape mechanisms, therapy resistances, the tumor microenvironment (TME) and epigenetics, which represents the rational for new preclinical and clinical investigations resulting in the implementation of novel immunotherapeutic treatments. Since immunotherapies in different solid tumors have only a modest benefit for the patients, an improved efficacy of immunotherapies is urgently needed. This requires a better knowledge about tumor immune surveillance and evasion as well as processes involved in acquired therapy resistances as well as possible combinations of agents restoring the immune escape phenotype. Therefore, in depth analyses of the host immune responses might represent the basis for the design of novel therapeutic interventions as well as combinations of current immunotherapies with other treatment options including agents targeting complementary mechanisms. However, the identification of new possible treatment modalities requires further laboratory-based discovery work including different “omics”-based high throughput analysis and bioinformatics tools including artificial intelligence. These experimental strategies might help to search for the most innovative and promising combination partners of immunotherapies, such as small targeted drugs or monoclonal antibodies (mAbs), which have the capacity to, e.g., overcome the immune suppressive TME or revert tumor evasion mechanisms, but the sequence and selection of drug combinations have still to be determined. Monitoring of the patients’ immune status prior and during therapy might help identifying the mode of treatment.

This annual TIMO meeting is a forum of experts from basic science and clinicians, which allows to discuss the current and future developments in basic and clinical research of tumor immunology, immunotherapy and immune oncology topics. During the recent years, an increased understanding why certain tumor types respond to immunotherapy, in particular immune checkpoint inhibitors (ICPis), has been obtained. This led to the assumption that novel treatment concepts with new agents and/or combinations are required to prevent or overcome immune resistance. A major pre-requisite for understanding the biology of resistance mechanisms is an increased knowledge of the tumor immune surveillance and escape, which then allow identifying new biomarkers, which could be used for monitoring of the success of novel treatment options.

TIMO XVI is composed of a comprehensive workshop to promote young investigators and a symposium program covering all different topics described above. These include in particular the role of microRNAs (miRNAs) in reversing immune escape mechanisms, epigenetic reprogramming, novel therapeutics targeting e.g. GDF-15, known as a cytokine with strong immune modulatory capacity, as well as changes in the tumor and immune metabolism, which affect immune suppression and response to immunotherapies. In addition, technological advances applied for immune monitoring were presented.

The symposium started with a talk of the organizer, *Barbara Seliger* (Halle, Germany), who summarized novel insights into different immune evasion strategies of cancer cells with a major focus on the downregulation or loss of the HLA class I antigen processing machinery (APM) components. These were frequently found in different tumor types and are associated with a reduced survival and resistance of cancer patients to different immunotherapeutic approaches. The underlying mechanisms of such HLA class I alterations could occur at distinct levels including posttranscriptional regulation, which is often mediated by a deregulation of immune modulatory miRNAs (im-miRs) targeting immune-relevant molecules. These im-miRs, mainly identified by the Seliger team using a miRNA enrichment technology in combination with RNA sequencing, control different HLA class I APM components as well as the immune checkpoints (ICPs) HLA-G and PD-L1, which are associated with the disease outcome and the cellular composition of the TME. Furthermore, other factors, like members of the small leucine-rich proteoglycan family, like biglycan (BGN), were downregulated in tumors and oncogene-transformed cells when compared to corresponding non-neoplastic controls, which could be reverted by restoration of BGN expression. Furthermore, high BGN expression levels directly correlated with increased HLA class I APM component expression, which was partially due to a downregulation of the oncogenic miR-21, an increased expression of APM components and an increased overall survival (OS) of patients.

*Andrea Anichini* (Milan, Italy) focused his talk on diverse markers and transcription factors (TFs) involved in melanoma differentiation in association with the phenotypic plasticity. Upon comparison of the expression of melanoma differentiation antigens and immune-related signatures in TCGA datasets of differentiated versus less differentiated tumors, he demonstrated an inverse correlation of genes linked to melanocytic differentiation versus genes involved in the mesenchymal status, which was associated with therapy response or resistance. While MITF^low^ melanoma cells were resistant to MAPK and ERK inhibitors, this was inversely correlated with Wnt5a and ZEB1 expression. The TF NFATc2 expressed in MITF^low^ melanoma cells was linked to an epithelial–mesenchymal transition (EMT)-like program of melanoma cells associated with myc, FOX1 and EZH2 expression. Targeting NFATc2 by si/shRNA or by an inhibitor known to downregulate EMT-related markers inhibited the migratory and invasive capacity of melanoma cells in vitro and in vivo, which is caused by an altered expression of downstream molecules thereby reversing the EMT-like program.

*Jan Budczies* from the University Hospital Heidelberg, Germany, discussed the composition of the TME of lung cancers upon ICPi therapy of long-term responders and non-responders (rapid progressors) by Nanostring-based gene expression profiling and by immunohistochemistry (IHC). A benefit of the ICPis in relation to 14 different immune cell populations and gene expression was found, which allowed to distinguish cold (no or low immune cell infiltration) and hot (high immune cell infiltration) tumors. Increased progression-free survival and long-term response to ICB was positively associated with the abundance of B cells, CD45^+^ cells and macrophages in the TME. He suggested the use of B cells together with the overall lymphocyte infiltration as a prognostic marker for ICPi response and the integration of gene expression profiling into the diagnostic workflow of lung cancer biopsies in order to select patients undergoing immunotherapy. By comparing the immune cell repertoire in lung adenocarcinoma driven by ALK fusions and lung adenocarcinoma driven by by EGF-R mutations distinct mechanisms of immune suppression were identified with a higher abundance of regulatory T cells (Tregs) in ALK^+^ tumors and a lower abundance of cytotoxic T cells in EGF-R^+^ tumors.

*John Trowsdale* from the University of Cambridge, Great Britain, demonstrated a high variation of the NK cell receptor interaction with HLA molecules dependent on ethnicities killer Ig-like receptor (KIR) types of more than 20.000 samples and demonstrated novel structural variations with KIR gene haplotype variations across different populations, e.g. Europeans and populations from Gambia and Uganda. Since some KIR alleles were identical, but exhibit different disease associations, a variation outside of the KIR coding region was suggested, such as the disruption of the TF binding sites in the anti-sense promoter thereby resulting in an increased transcription. Extreme variations of HLA/KIR combinations can drive autoimmune diseases, infections, cancer as well as pregnancy, including pre-eclampsia. For example, in Uganda, 10% of pregnancies are linked to pre-eclampsia, but only 3% in Europe. Furthermore, KIR / HLA interactions are associated with resistances to malaria in Uganda thereby regulating the NK cell-mediated production of cytokines and direct cytotoxic killing of parasitized red blood cells.

Based on the unresponsiveness of a subset of patients to ICPi and a heterogeneous HLA class I expression of tumors, *Adelheid Cerwenka* from the University of Mannheim, Germany, suggested that additional effector cells, like NK cells, should be exploited. However, there exist some major challenges for NK cell-based therapies in solid tumors, since NK cells are short lived and are often non-functional, which is due to changes of the TME. Hypoxia accompanied by HIF1α expression has been demonstrated to impair NK cell activation and effector function. Inhibition of HIF1α enhanced NK cell-mediated cytotoxicity, which is driven by the aryl hydrocarbon receptor (AHR) resulting in responsiveness to IL-12, IL-18, NF-kB activation, regulation of metabolism, mTOR pathway activation as well as mitochondrial fitness. Thus, AHR primes for higher responsiveness and activation of HIF1α-deficient NK cells under hypoxia.

The implementation of NK cells as therapeutics was addressed by *Ulrike Köhl* from the Fraunhofer Institute for Cell Therapy and Immunology in Leipzig, Germany. She reported on chimeric antigen receptor (CAR) effector cells and their function to specifically attack and kill cancer cells via activation of effector function using antigen receptors. Despite some advanced therapeutic medicinal products have been approved and demonstrated an outstanding benefit of, e.g., autologous CD19^+^ CAR T cell treatment, disadvantages are severe side effects of the treatment, a frequent failure of complete long-term remission and less success in solid tumors. Since the manufacturing of personalized CAR T cells is time-consuming, a monitoring of patients and a quality control of manufacturing to avoid failure of CAR T cell production are urgently needed. It requires biomarker identification based on “omics” technologies including RNA-seq and advanced flow cytometric analyses, which should contribute to an optimization and harmonization of the manufacturers’ protocols. However, a switch to allogenic effector cells, in particular NK cells has some advantages, like the lack of severe adverse side effects in patients as well as the lack of graft versus host disease (GVHD). Previous clinical trials demonstrated that IL-2 stimulation was associated with an improved NK cell cytotoxicity, while an impaired NK cell cytotoxicity in tumor patients can be mediated by immune escape mechanisms. Prof. Köhl summarized these results in the context of the first use of human CAR NK cells in patients with relapsed or refractory B lymphocyte malignancies resulting in a high frequency of complete or partial remission in the absence of GVHD and toxicity. In addition, first trials using the combination of CAR NK cells with anti-PD-L1 mAb seemed to further improve response rates. Thus, allogeneic CAR NK cells might have the power as an additional tool regarding future treatment of cancer patients.

The talk by *Marco Gerlinger* and co-authors from the Barts Cancer Institute in London, United Kingdom, described how patterns of cancer evolution define immunologically distinct subgroups of mismatch repair deficient (MMRd) colorectal cancers (CRCs). Between 40 and 60% of MMRd CRCs respond to immunotherapy with ICPis and these new data may lead to novel insights into resistance mechanisms and enable the development of predictive biomarkers. Multi-region sequencing showed that truncal driver aberrations were common in the TGF-β receptor 2, BRAF, ERBB2 and APC. In contrast, driver mutations that confer immune evasion, such as inactivating mutations in the MHC antigen presentation pathway (β_2_-microglobulin, TAP1, NLRC5) or of the interferon-γ signaling pathway (JAK1) showed a high intra-tumor heterogeneity and often parallel the evolution of multiple distinct immune evasion drivers in different parts of the same tumor. Tumors with pan-tumor inactivation of these pathways had low CD8^+^ T cell infiltrates indicating a successful immune evasion, whereas those with subclonal evolution showed high T cell infiltrates supporting ongoing evolution due to immune selection pressures. Surprisingly, tumors without any genetic immune evasion drivers showed low CD8^+^ T cell infiltrates supporting the activity of a still unknown mechanism of immune evasion. The high intra-tumor heterogeneity likely explains why the development of predictive biomarkers for ICPis from single biopsies has so far been unsuccessful in MMRd CRCs. It shows the need for biomarkers that can resolve clonal and subclonal immune evasion drivers and then assign individual cancers to the three evolution groups (pan-tumor, subclonal and absence of immune evasion). Circulating tumor DNA testing can be readily implemented in the clinic for this purpose and this is being tested in follow on studies.

*Oliver Kepp* from INSERM in Paris, France, focused his talk on immunogenic cell death (ICD) inducers and enhancers, which act on cancer and dendritic cells (DC), respectively. An unbiased high throughput chemical compound screening led to the identification of crizotinib as a novel ICD inducing tyrosine kinase inhibitor. Crizotinib increased the efficacy of the non-immunogenic chemotherapeutic cisplatin in vitro and in several orthotopic non-small cell lung carcinoma (NSCLC) models in mice. The effect of crizotinib was abolished by T cell depletion or interferon (IFN)-γ neutralization. Further sequential combination with mAbs targeting PD-1 and CTLA-4 resulted in 80% cure of orthotopic lung cancers in vivo underlining the sensitizing effect of crizotinib-mediated ICD induction for subsequent ICPi.

*Michele Maio* from the University Hospital in Siena, Italy, gave novel insights regarding the treatment with ICPi alone or in combination with demethylating agents of diverse cancers, but in particular of melanoma. The combination treatment of the ICPi nivolumab and ipilimumab to a single ICPi increased the survival of advanced melanoma patients. However, not all patients had an improved OS and some patients lack response to this therapeutic option due to either primary or secondary resistances. Since these might be mediated by changes in the TME, targeting of the TME as well as of the tumor itself could enhance the ICPi efficacy, which might be also epigenetically controlled. Indeed, non-mutational epigenetic reprogramming has been recently suggested as a hallmark of cancer. Early work from Dr. Maio’s group demonstrated an upregulation of the expression of HLA class I antigens, tumor antigens, components of the interferon (IFN) pathway and co-stimulatory molecules in tumor cells upon treatment with the demethylating agent 5-aza 2‘-deoxycytidine (DAC) thereby inducing CTL recognition. Treatment of tumor cell lines with distinct mutational phenotypes with various epigenetic drugs was associated with an altered expression of immune-relevant molecules. This lead to the hypothesis that that epigenetic immune remodeling of neoplastic cells could be used to overcome resistances to ICPi therapy, which is currently in a novel trial combining ICPi with DAC treatment.

*Nathalie Labarriére* from INSERM in Nantes, France, reported on adoptive T cell therapy (ACT) in melanoma by infusion of melanoma-specific T lymphocytes to prevent the relapse of treated patients with focus on the two melanoma specific antigens MelanA and the MELOE-1. Melan-A and MELOE-1 specific T cells sorted from patient blood and amplified are highly reactive against their cognate peptides and HLA-A2 melanoma cells. A significant fraction of these T cells express high levels of PD-1 that could decrease their anti-tumor functions in the TME. Therefore, Labarriére and colleagues edited PDCD1 melanoma-specific T cells and monitored their anti-tumor activity upon adoptive transfer in immunodeficient mice. RNA-seq analysis revealed that PDCD1-edited T cells underexpressed a panel of genes related to cell cycle and proliferation, while exhibited a sustained expression of TIGIT, another ICP closely linked to PD-1. These results highlighted the complexity of ICP edition for therapeutic T cells, and the need to define the better target for such genome modification.

The intestinal microbiome, a novel hallmark of cancer, and its role in cancer immunotherapy, was addressed by *Marcel van den Brink* from the Memorial Sloan Kettering Cancer Center in New York, USA. The outcome of allogeneic hematopoietic stem cell transplantation (HSCT) concerning OS, infection, GVHD, organ toxicity as well as relapse has been associated with different gut bacteria. Analysis of more than 8.500 samples obtained from more than 1.300 HSCT recipients from different transplantation centers demonstrated an association of low intestinal microbiota diversity at neutrophil engraftment with a decreased OS. Enterococcus in the gut flora increases the risk of acute GVHD and reduces survival due to enterocyte damage and dysbiosis. Since antibiotics, conditioning regiments, diet, but also other drugs could affect the intestinal microbiota composition, mechanistic analyses using preclinical models were performed. The exposure to a broad spectrum of antibiotics is associated with GVHD mortality and might be due to dysbiosis, immune modulation and colon epithelial damage. A meta-analysis of large cohorts consisting of cancer patients treated with ICPs and antibiotics demonstrated a negative association of antibiotic administration with the efficacy of ICPs. Furthermore, the exposure of antibiotics four weeks prior to CAR T cell therapy was associated with a decreased OS in CD19^+^ hematological malignancies. Analyses of microbiome clusters overtime in intestinal microbiota of HSCT patients upon non-antibiotic drug exposure demonstrated an increased number of enterococcus relative abundance in allogeneic HSCT patients, which was predictive for the clinical outcome following allogeneic HSCT. In addition, a role of bile acid (BA) metabolism in modulation of T cells was demonstrated in GVHD mice, which have a reduced amount of total primary and secondary BA and a reduced BA metabolism in the liver accompanied by low abundance of anaerobe commensal bacteria and an extension of enterococcus and proteobacteria. Since primary BA are potent agonists of the farnesoid receptor (FXR) and FXR expression is induced upon activation of naive CD8^+^ T cells, the FXR activity should be modulated by drugs or the enhancement of BSH/secondary BAs to enhance OS of HSCT patients. Changes in the intestinal flora are associated with OS, lethal GVHD, bacteria load/ sepsis, engraftment and relapse in allo- and autologous stem cell transplantation. Broad spectrum antibiotics are associated with worse outcomes in allo-HSCT and CAR T cell-treated patients. Furthermore, GVHD was associated with low levels of secondary BA and unconjugated BA, while FXR deficiency in donor T cells result in less GVHD.

*Stefan Glück* provided a general overview on the current state of the art as well as regarding the future of immune oncology suggesting a paradigm shift in cancer therapy targeting tumor cells to immune and non-immune cells, based on the increased knowledge of the TME composition. The major aspects of anti-tumoral immune responses regulated by B cells, T cells, macrophages/ monocytes as well as immune suppressive cells like regulatory T cells (Treg) and myeloid-derived suppressor cells (MDSC) were summarized. Next to the role of immune cell subpopulations, checkpoints (CPs), such as cell cycle CPs, Krebs cycle CPs, immune cycle CPs and others, as targets for cancer were addressed. In addition, the importance of the tumor mutational burden (TMB) defined regarding the survival of patients and response to ICP was discussed. Three categories of CP responses were identified ranging from stable disease, partial response to total response, which depend on the tumor entities with high responders for squamous cell cancer, non-small cell lung cancer, renal cancer, bladder cancer as well as melanoma, while non-responders include in particular glioblastoma, prostate cancer, pancreatic cancer as well as microsatellite stable CRC. The patients’ categorization was based on the immune cell infiltration of the tumor characterized by preexisting immunity, functional immune response, a totally immune excluded infiltrate as well as an immune desert. In addition, chemotherapy could provide stimulatory effects on different parameters of the immune system, like an upregulation of T cell function, Th1 polarization, secretion of pro-inflammatory cytokines, antigen presentation and epitope spreading, an increased tumor antigen and danger release, but a downregulation of Tregs and MDSC. Combination of chemotherapy with ICPi demonstrated an increased clinical activity and an improved survival of patients, but the number of long-term survivors was limited. Challenges for increased response rates were non-immunogenic tumors, resistance development, severe side effects and lack of reliable biomarkers for distinguishing responders from non-responders.

*Sylvia Adams* from the New York University School of Medicine and Director of the Breast Cancer Center, USA, reported about the modulation of anti-tumor responses to improve the survival of BC patients. During the last years, BCs have been shown to be heterogeneously infiltrated with CD4^+^ and CD8^+^ T cells, but also with tertiary B lymphocyte structures, which are surrogates of effector immune responses. Furthermore, differences in the percentage of stromal tumor-infiltrating lymphocytes (TILs) exist between distinct BC subtypes. In triple-negative breast cancer (TNBC), the number of TILs has been directly associated with disease outcome and has been suggested as a prognostic marker, but also as predictive biomarkers for chemotherapy response. In the Key Note-0862 trial using pembrolizumab, anti-tumor activity in previously untreated PD-L1^+^ patients demonstrated an increased survival upon pembrolizumab treatment. Combination trials with ICPis or chemotherapy with PD-1/ PD-L1 monoclonal antibodies (mAbs) lead to an increased OS of BC patients. Thus, the therapy of stage I TNBC could be optimized dependent on the frequency of stromal TILs, which represented the rational for the design of a clinical trial, where the monitoring of the immune cell infiltration will be a measure for patients’ randomization.

*Miriam Molina-Arcas* from the Crick Institute in London, Great Britain, talked about the impact of the KRAS oncogene signaling on the TME with focus on lung cancer, KRAS as the most common driver oncogene with a frequency of 32% in this disease. KRAS-G12C inhibitors have been developed and recently implemented in phase II clinical trials demonstrating impressive results. Since the development of resistances limits the duration of response, a need of combination therapies with inhibitors of pathways involved in tumor immune evasion by KRAS is suggested. Tumor adaptation to targeted agents thereby developing drug resistances has also been demonstrated for a number of combinations of tyrosine kinase inhibitors (TKIs) with chemotherapeutics. It has become evident that oncogenes and tumor suppressor genes can shape the immune cell composition of the TME, but also the secretion of diverse modulatory factors thereby negatively interfering with immune responses. In a murine lung tumor model, KRAS inhibition reduced the myeloid cell infiltration and increased the antigen presentation as well as IFN responses and T cell infiltration and activation, in particular in combination with IFN-γ due to an increase in effector memory cells, but these T cells were more exhausted. In addition, KRAS promotes changes in the macrophage polarization and downregulates monocyte and neutrophil recruitment. Treatment of “cold” tumors with combinations of KRAS inhibitor and ICPis, such as anti-PD-1, demonstrated no synergy, while an increased sensitivity and anti-tumoral immunity was demonstrated in “hot” tumors. Furthermore, tertiary lymphoid structures (TLS), which target endogenously retroviruses and are protective in vivo, are predictive for response to immunotherapy as demonstrated by a stimulation of anti-tumor B cell responses upon KRAS inhibition.

*Lorenzo Galluzzi* from the Weill Cornell Medical College in New York, USA, harnesses an innovative mouse model of hormone receptor (HR)-positive breast cancer with unique translational potential and determine immunological mechanisms of resistances to CDK4/6 inhibitors, a novel class of targeted anti-cancer agents that have been shown to extend the OS of patients with HR^+^ breast cancer. Besides operating as cytostatic agents, CDK4/6 inhibitors mediate a variety of immunomodulatory effects that may indeed participate in their clinical activity. The authors previously demonstrated that radiation therapy can enhance the therapeutic effects of CDK4/6 inhibitors in preclinical models of HR^+^ and TNBC, but only when administered according to a specific treatment schedule. Novel, unpublished data from the team suggest that *γδ* T cells may be involved in the resistance of patients with HR^+^ breast cancer to CDK4/6 inhibitors.

Known signatures of tumors upon treatment with radiotherapy in combination with immunotherapy were analyzed by *Udo Gaipl* from the Department of Radiation Oncology, University Hospital in Erlangen, Germany, in order to determine their role in prediction of responses to solid tumors. Local radiotherapy causes ICD, necrosis, release of poor inflammatory cytokines as well as the expression of immunogenic cell surface molecules, which induces anti-tumor immune response. This is mediated by the release of DAMPs and antigens, which are uptaken and presented by DCs and allow T cell priming and recognition of tumor cells by CD8^+^ T cells. Radiotherapy in combination with anti-PD-1 therapy affects immune cell infiltration, local and abscopal tumors, but treatment schedules including the dose have to be optimized, since the damage on the TME could impair therapy response. In this context, cancer-associated fibroblasts play a role, which then can lead to senescence and accumulation in the extracellular matrix generating therapy resistance. One pre-requisite for biomarkers to predict response is an immune monitoring approach of PBMCs using multicolor flow cytometry. Changes in the innate and adaptive immune cells were found by monitoring a phase III trial on NSCLC and HNSCC, which could serve also as predictive to therapy response. However, dynamics of the immune changes have to be considered to define stable and immune markers for prediction. Treatment of locally advanced HNSCC with a combination of inhibitors, radiotherapy and chemotherapeutics demonstrated a high pathological clinical response with manageable related toxicity effects and immune markers associated with treatment response or failure. In conclusion, the (immunological) outcomes of patients are dependent on the dose of RT and on combinations with immunotherapy and targeting of lymph nodes.

*Veronica Huber and Elena Daveri* from Fondazione IRCCS Istituto Nazionale dei Tumori, Milan, Italy, talked about novel strategies for functional reprogramming myeloid-derived suppressor cells (MDSC) in cancer patients. Indeed, this heterogeneous cell subset associated with poor disease outcomes and response to treatment in multiple malignancies represents a still unexploited target of immunomodulation in cancer therapy. The INT team has been addressing the topic by developing a simplified Myeloid Index Score, i.e., a 4-marker (CD15, CD14, HLA-DR and PD-L1) flow cytometry panel to be applied in PBMC, which clearly predicts prognosis in advanced melanoma patients independently of the type of treatment (BRAF/MEK, TKI *versus* ICPis). Dissecting the molecular properties of tumor-conditioned CD14^+^ cells, a panel of specific miRNAs was found to associate with the immunosuppressive and pro-inflammatory functions of MDSCs and to predict resistance to ICPi, but not TKI in melanoma patients. Furthermore, PLGA nanoparticles (NPs) embedded with antagonists of these miRNAs were able to blunt the ex vivo activity of patient-derived MDSCs, whereas NPs with miRNA-mimics turned monocytes into bonafide MDSCs in vitro*.* NPs were also able to mediate MDSC reprogramming when cargoed with proton pump inhibitors (esomeprazole) and anti-angiogenic TKI (cabozantinib). Finally, the INT group evaluated whether cancer-associated MDSC accrual could be contrasted by short-term life style changes in clinical setting. Data generated in patients with breast cancer and Familiar Adeno-Polyposis demonstrated that this regimen (i) reshapes the pro-inflammatory myeloid systemic pool, (ii) decreases resident CD68^+^ cells and (iii) boosts T cell and NK cell immunity both in the periphery and at the tumor site. Thus, MDSC can be targeted by both drug-based and dietary interventions. This results in a significant recovery of immune effector functions, which could be exploited by combination therapies in a patient-tailored manner.

*Douglas McNeel* from the University of Wisconsin in Madison, USA, gave insights into the development of DNA vaccines for the treatment of prostate cancer. Despite the FDA-approved first anti-tumor vaccine Sipuleucel-T resulted in an improved OS, its efficacy was low suggesting combination approaches targeting mechanisms of resistance for optimal therapeutic vaccine strategies. Furthermore, anti-tumor vaccines might be more effective in minimal residual disease prior to the tumor development of multiple means of resistance. A DNA vaccine encoding prostatic acid phosphatase (PAP) was developed and a phase II trial comparing vaccine with granulocyte–macrophage colony-stimulating factor (GM-CSF) or GM-CSF alone demonstrated no overall increased time to disease progression. However, a combination of PD-1 blockade with vaccination demonstrated PSA response in patients and an induction of PAP-specific immunity associated with a decline of PSA levels and increased tumor infiltrating CD8^+^ T cells. About 42% of patients developed adverse grade 1 and 2 effects upon this combinatorial treatment, which was associated with greater time to progression. Based on these promising results, novel trials evaluating DNA vaccines in combination with ICPis are currently underway, such as a recently started randomized phase II clinical trial combining ICP antibodies with one or two vaccines after castration resistance, and a neoadjuvant trial in patients with high-risk prostate cancer prior to prostatectomy. In the composition of the TME, treatment resulted in increased CD8^+^ T cells recruitment and activation. Since CD8^+^ T cell activation results in the expression of multiple T cell receptor checkpoints, a combination of DNA vaccines with PD-1 and LAG-3 blockade was implemented. Furthermore, the role of toll-like receptor (TLR) activation on PD-1 expression at the time of T cell activation was determined. TLR agonist stimulation as a vaccine adjuvant improved anti-tumor immunity and combinations affect the expression of different T cell checkpoints. Using combinations of TLR agonists with DNA vaccines reduced the growth of prostate cancer cells in a murine prostate cancer model suggesting that vaccines could be used as T cell activators in combination with activation modifying agents, which might improve anti-tumoral responses.

*Peter Siska* from the University Hospital in Regensburg, Germany, talked about kynurenine (kyn)-induced metabolic effects on T cells in different cancer types in association to inflammation. Indolamine 2,3-deoxygenase (IDO) degrades tryptophane to produce kyn, and tryptophane depletion as well as kyn can cause immune modulation in tumors. Therefore, the project group investigated the effect of kyn on T cell activity and its underlying mechanisms as well as the use of kyn to inhibit auto- and alloreactivity in vivo. Interestingly, increased levels of kyn were found in tumor tissues compared to non-tumorigenic control tissues. Yet, the levels did not reach those required for in vitro T cell inhibition, thus potentially explaining the failure of IDO inhibition in a phase III trial. Nevertheless, exogenous kyn induced suppression of T cells in vitro, which was mediated by AHR and by depletion of intracellular fatty acids. The use of kyn for inhibition of auto- and alloreactivity was demonstrated by adoptive transfer in colitis and heart transplantation models. Thus, kyn might suppress inflammation in vivo, which should be further tested.

*Sergio Rutella* from Nottingham Trent University in Great Britain talked about an immune dysfunction gene signature in acute myeloid leukemia (AML) postulating that immune exhaustion and senescence are major hallmarks of dysfunctional CD8^+^ T cells and of prognostic relevance in AML. He demonstrated that senescent-like bone marrow (BM) CD8^+^ T cells are not able to eliminate AML blasts in vitro, which was directly correlated to worse patient survival. Using bulk RNA-seq and single-cell RNA-seq data, he identified novel immune dysfunction signatures in newly diagnosed, chemotherapy-naive AML, which were associated with prognostic molecular alterations, including *TP53* mutational status, phenotype, and poor patient outcome. Furthermore, the newly defined immune landscapes predicted chemotherapy resistance as well as response to checkpoint inhibitors pembrolizumab and nivolumab in relapsed/refractory AML. This analysis leveraged broad cohorts of primary BM samples from children and adults with AML, demonstrating an age-dependence of an IFN-γ-related mRNA profile and molecular disease subtypes, which were predictive for therapy resistance or response. This approach does not only give novel insights into the immune biology of AML, but also provides the rational for personalized (immuno) therapies in AML subtypes.

Despite the large number of interesting talks during the symposium demonstrating an increased knowledge in the field of tumor immunology and immunotherapies, it becomes obvious that a number of urgent topics have still to be addressed. These include single-cell RNA sequencing as well as spatial omics studies to get in depth insights into the TME and its heterogeneity regarding tumor-specific spatial patterns of immune cells. Although patterns of immune evasion and evolution as key determinants of immunogenicity correlated with the CD8^+^ T cell densities, the analysis of a single-cell population is insufficient for immunotherapy prediction and also demands for novel biomarker development, such as, e.g., cell-free tumor DNA. To avoid tumor immune escape, multiple targets, more specific immunogenic shared antigens and modulation of the immune suppressive TME by gene editing strategies targeting the inhibitory ligands have to be implemented in the future. To increase therapeutic efficacy, therapies using T, B, NK cells and macrophages should be developed and ICPis used in combinations with other treatment modalities, such as, e.g., different immunotherapeutics, chemotherapy, radiation therapy and targeted agents, but also combinations with the microbiome, antibiotics treatment and viral approaches have to be taken into account. Peripheral blood-based immune signatures predicting treatment outcomes should be developed also in the view of the dynamic changes in immune cell markers, cell composition and responses.

## Abbreviations

ACT: Adoptive cell therapy
AHR: Aryl hydrocarbon receptor
ALL: Acute lymphatic leukemia
AML: Acute myeloid leukemia
APC: Antigen presenting cells
Arg1: Arginase-1
β_2_-m: Beta-2-microglobulin
BA: Bile acid
BGN: Biglycan
BM: Bone marrow
CAR: Chimeric antigen receptor
CPI: Checkpoint inhibitors
DAC: 5-Aza 2‘-deoxycytidine
DC: Dendritic cells
DLBCL: Diffuse large B cell lymphoma
ECM: Extracellular matrix
EGFR: Epidermal growth factor receptor
FDA: Federal rug Agency
FXR: Farnesoid receptor
GM-CSF: Granulocyte–macrophage colony-stimulating factor
GMP: Good medical practice
GVHD: Graft versus host disease
HC: Heavy chain
HCT: Hematopoietic cell transplantation
HIF: Hypoxia inducible factor
HLA: Human leukocyte antigen
HNSCC: Head and neck squamous cell cancer
HR: Hormone receptor
HSCT: Hematopoietic stem cell transplantation
ICB: Immune checkpoint blockade
ICD: Immunogenic cell death
ICP: Immune checkpoint
ICPi: Immune checkpoint inhibitor
IDO: Indolamine 2,3-deoxygenase
IFN: Interferon
IHC: Immunohistochemistry
iNOS: Inducible nitric oxide species
KIR: Killer Ig-like receptor
Kyn: Kynurenine
mAb: Monoclonal antibody
MDSC: Myeloid-derived suppressor cell
miRNA: MicroRNA
MMRd: Mismatch repair deficient
MMRp: Mismatch repair proficient
NGS: Next generation sequencing
NP: Nanoparticle
NSCLC: Non-small cell lung carcinoma
OR: ODD ratio
OS: Overall survival
PAP: Prostatic acid phosphatase
PBL: Peripheral blood lymphocytes
PD1: Programmed death receptor 1
PD-L1: Programmed death ligand 1
PFS: Progression-free survival
RCC: Renal cell carcinoma
RR: Response rate
SLN: Sentinel lymph node
TAN: Tumor associated neutrophils
TCR: T cell receptor
TF: Transcription factor
TIL: Tumor infiltrating lymphocyte
TKI: Tyrosine kinase
TLR: Toll-like receptor
TLS: Tertiary lymphoid structures
TME: Tumor microenvironment
TNBC: Triple negative breast cancer
Treg: Regulatory T cell

